# Erectile dysfunction and quality of life in men receiving methadone or buprenorphine maintenance treatment. A cross-sectional multicentre study

**DOI:** 10.1371/journal.pone.0188994

**Published:** 2017-11-30

**Authors:** Fabio Lugoboni, Lorenzo Zamboni, Angela Federico, Stefano Tamburin, for the Gruppo InterSERT di Collaborazione Scientifica (GICS)

**Affiliations:** 1 Department of Medicine, Addiction Medicine Unit, Verona University Hospital, Verona, Italy; 2 Department of Neurosciences, Biomedicine and Movement Sciences, University of Verona, Verona, Italy; Northwestern University, UNITED STATES

## Abstract

**Background:**

Erectile dysfunction (ED) is common among men on opioid replacement therapy (ORT), but most previous studies exploring its prevalence and determinants yielded contrasting findings. Moreover, the impact of ED on patients’ quality of life (QoL) has been seldom explored.

**Objective:**

To explore the prevalence and determinants of ED in men on ORT, and the impact on QoL.

**Methods:**

In a multicentre cross-sectional study, we recruited 797 consecutive male patients on methadone and buprenorphine treatment, collected data on demographic, clinical, and psychopathological factors, and explored their role as predictors of ED and QoL through univariate and multivariate analysis. ED severity was assessed with a self-assessment questionnaire.

**Results:**

Nearly half of patients in our sample were sexually inactive or reported some degree of ED. Some demographic, clinical and psychopathological variables significantly differed according to the presence or absence of ED. Multivariate regression analysis indicated that age, employment, smoke, psychoactive drugs, opioid maintenance dosage, and severity of psychopathological factors significantly influenced the risk and severity of ED. QoL was worse in patients with ED and significantly correlated with ED severity. Age, education, employment, opioid maintenance dosage, ED score, and severity of psychopathology significantly influenced QoL in the multivariate analysis.

**Conclusions:**

ED complaints can be explored in male opioid users on ORT through a simple and quick self-assessment tool. ED may have important effects on emotional and social well-being, and may affect outcome.

## Introduction

Erectile dysfunction (ED) is defined as the inability to achieve or maintain an erection satisfactory for the completion of sexual activity [1]. ED is a prevalent health condition, which is estimated to affect 17.7–18.4% men aged ≥ 20 years in the USA [2,3] and 32.2% in Europe [4], may be episodic or chronic, and can be associated with other sexual dysfunctions including reduced sexual desire and premature ejaculation [5]. Common risk factors for ED encompass age, obesity, diabetes, hyperlipidemia, lower urinary tract symptoms, hypertension, low physical activity, smoke [2,4], psychiatric conditions and psychological factors [5].

Opioids may increase sexual desire in the short term [6], but their long term use is known to negatively impact on sexual function and to lead to erectile dysfunction (ED) [7,8]. Various studies explored ED in men on opioid replacement therapy (ORT) and reported a 21–52% prevalence [7,9–11], with a peak of approximately 80% in a longitudinal study on a small group of patients in China [12]. Most studies were on small samples of men on ORT, but a meta-analysis suggested that factors associated with sexual dysfunction include age, familial status, medical comorbidity, psychiatric illness, testosterone levels, opioid dosage, duration of treatment, and other current substance use disorders [13].

The majority of studies on ED were conducted on men under methadone maintenance treatment, and a meta-analysis indicated a 52% pooled prevalence for sexual dysfunction among methadone users [11]. Buprenorphine was introduced more recently as an alternative ORT [8]. European data indicate that methadone is prescribed in approximately 63% of patients, while buprenorphine is prescribed in 37% of them, although these figures differ greatly from country to country [14]. There is agreement that substitution treatment, either methadone or buprenorphine, may improve ED in opioid users [6,15].

Pharmacodynamics of methadone and buprenorphine differ, in that the former is a μ-opioid receptor agonist, while the latter is a mixed agonist-antagonist opioid that acts on the μ-opioid receptor with low intrinsic activity and high affinity, and on the κ-opioid receptor with no intrinsic activity but high affinity [7,8].

Human and animal data indicate that opioids may act at different sites in the hypothalamic–pituitary axis, and lead to opioid associated androgen deficiency (OPIAD), an endocrine dysfunction that includes increase of prolactin and decrease of follicle stimulating hormone, luteinizing hormone, testosterone, estradiol, and oxytocin [16,17]. OPIAD results in hypogonadism, and is reported to occur after a few weeks of opioid intake and when exceeding a daily oral morphine milligram equivalent dosage (OMMED) of 100 mg [17]. Animal data suggest lower risk of OPIAD with buprenorphine than methadone probably through the offset of hypothalamic–pituitary axis inhibition related to κ-opioid receptor antagonist activity [18]. Data from humans are discordant, in that some studies reported more frequent sexual and ED to methadone, while other ones found no significant differences between the two maintenance treatments [7–9,19,20].

Sexual functioning is an important determinant of quality of life (QoL), but the effect of ED on QoL has been seldom explored in men under ORT [8,21,22].

The aims of the present cross-sectional multicentre study are to explore the prevalence and severity of ED in patients under methadone or buprenorphine maintenance treatment, the factors related to ED, and the impact of ED on patient’s QoL. To this aim, we recruited a large sample of patients, collected data on a number of demographic, clinical, and psychopathological factors, and explored their role as predictors of ED and QOL through univariate and multivariate analysis.

## Materials and methods

Patients were recruited from November 1^st^, 2014 to January 31^st^, 2015 in twenty-two National Health Service Drug Addiction Units that belong to the *Gruppo InterSERT di Collaborazione Scientifica* (GICS), a scientific collaborative network dealing with drug-related problems and located in Italy. The inclusion criteria were: a) male sex, b) age 18 years or older, c) history of opioid use, d) having been on oral methadone or oral buprenorphine treatment for at least 6 months, e) absence of any relevant comorbidity that might have influenced erection and/or QoL, including diabetes mellitus, peripheral vascular disease, neurological diseases that may be associated with ED (stroke, Parkinson’s disease, multiple sclerosis, spinal cord injury), severe obesity (i.e., body mass index > 35), drugs that might interfere with sexual function including hormonal treatment for prostate cancer or benign prostatic hypertrophy, urological conditions or previous surgical procedures that might cause ED, cancer, severe heart, lung, liver or kidney disease. Written informed consent was obtained prior to inclusion in the study, which was conducted in accordance with the declaration of Helsinki and approved by the Ethics Committee of the Verona University Hospital. No benefit was provided for participation in the study that was voluntary and confidential.

Demographic (age; education: grade school, high school, university; employment: unemployed, employed; marital status: single or divorced, engaged or married; parenthood: no, yes), and clinical variables (type of opioid: methadone, buprenorphine; daily OMMED: mg; smoke: yes, no; cigarettes: cigarettes/day; psychoactive drugs: yes/no; type of psychoactive drug: benzodiazepine, antidepressants, antipsychotics, more than one) were derived from clinical records. OMMED was calculated using standard dosage conversion calculations, i.e. a conversion factor of 4.7 for methadone and of 37.5 for oral buprenorphine [23].

Psychopathological symptoms were measured with the Symptom Check List-90-R (SCL-90-R), a widely-used 90-item scale that includes a number of different subscales exploring the severity of respondents’ symptoms over the previous seven days [24]. Each SCL-90-R item is rated on a 5-point Likert scale ranging from ‘not at all’ (0) to ‘extremely’ (4). We derived nine subscales, i.e., somatization (SOM), obsessive-compulsive (OC), interpersonal sensitivity (IS), depression (DEP), anxiety (ANX), hostility (HOS), phobic anxiety (PHO), paranoid ideation (PAR), and psychoticism (PSY), and the global severity index (GSI) from the Italian Version of the SCL-90-R [25]. The GSI, which was obtained by adding the scores of all 90 items and dividing by 90, is considered the single best indicator of the current level or depth of an individual's disorder, in that it combines information concerning the number of symptoms reported with the intensity of perceived distress [25].

Erectile function was explored with the abridged five-item version of the International Index of Erectile Function (IIEF-5), a self-administered questionnaire that explores quality of erectile function and sexual intercourse confidence and satisfaction [26]. Each IIEF-5 item is rated on a 5-point Likert scale, ranging from 1 to 5, with higher scores indicating better erectile function and satisfaction. The severity of ED (range: 5–25) was graded as none (IIEF-5 score: 22–25), mild (17–21), mild to moderate (12–16), moderate (8–11), and severe (5–7), and the absence of sexual intercourses was also recorded [26].

QoL was scored with the 12-Items General Health Questionnaire (GHQ-12), one of the most widely used screening tool, which measures changes in psychological health and is composed of 12 questions on mood states over the previous two weeks: lost sleep, feelings of being under strain, could not concentrate, felt unable to play a useful role, could not face problems, could not make decisions, could not overcome difficulties, felt unhappy, did not enjoy day-to-day activities, felt depressed, lost confidence, and felt worthless [27]. GHQ-12 items were scored on a 4-point scale, ranging from 0 to 3, with higher values indicating more severe psychological distress, and the overall score (range: 0–36) was calculated [27].

### Statistical analysis

All tests were carried with the IBM SPSS version 20.0 statistical package. The normality of variable distribution was analyzed with the Skewness-Kurtosis test. The Pearson’s χ^2^ test and Bonferroni’s corrected post-hoc with the non-parametric Mann-Whitney U test were applied to categorical variables. For continuous variables, the one-way ANOVA and post-hoc with Bonferroni’s correction were used in case of normal distribution, while the Kruskal Wallis H test and Bonferroni’s corrected Mann-Whitney U tests were applied if the distribution was not normal. The homogeneity of variances was analyzed with the Levene’s test, and the data were logarithmically transformed before submitting them to ANOVA if the variances were inhomogeneous. Correlations were explored with the Spearman’s ρ correlation coefficient. Multivariate analysis was used to explore the influence of the demographic and clinical covariates on ED and QoL measures. Linear regression model analysis was applied for IIEF-5 and GHQ-12 scores (continuous dependent variables). The risk of having ED was also explored with logistic regression model analysis (binary dependent variable: any ED, no ED), and the results were expressed as odd ratios and 95% confidence intervals (CI). The goodness of fit of the logistic regression model was assessed using the Hosmer and Lemeshow test [28]. *P* < 0.05 (two-tailed) was taken as the significance threshold for all the tests.

## Results

We recruited 1000 consecutive male patients. Demographic and clinical characteristics of those (N = 797, 79.7%), who answered the IIEF-5 questionnaire, are reported in Tables 1 and 2, respectively and the full dataset is attached as supporting information (S1 Dataset). In our cohort, 598 patients (75.0%) were taking oral methadone (average daily dosage: 58.0 ± 47.3 mg, median: 50, range: 2–350), and 199 patients (25.0%) were on oral buprenorphine (average daily dosage: 8.3 ± 6.7 mg, median: 6, range: 0.5–40).

**Table 1:** Demographic characteristics of the patients.

Variable	
Age (y)	mean: 38.6 ± 9.9, median: 39, range: 18–66
Education^‡^	493 (61.9%), 283 (35.5%), 21 (2.6%)
Employment^‡^	366 (45.9%), 431 (54.1%)
Marital status^§^	669 (83.9%), 128 (16.1%)
Parenthood^¶^	681 (85.4%), 116 (14.6%)

^†^Education: grade school, high school, university (%).

^‡^Employment: unemployed, employed (%).

^§^Marital status: single/divorced, engaged/married (%).

^¶^Parenthood: no, yes (%).

**Table 2:** Clinical characteristics of the patients.

Variable	
Smoke^†^	86 (10.8%), 711 (89.2%)
Cigarettes/day	mean: 15.2 ± 9.1, median: 15, range: 0–70
Psychoactive drugs^‡^	502 (63.0%), 295 (37.0%)
Antidepressants	57 (7.2%)
Benzodiazepines	148 (18.6%)
Neuroleptics	20 (2.5%)
More than one	70 (8.8%)
Opioid agonist^§^	598 (75.0%), 199 (25.0%)
Daily OMMED (mg)	mean: 282.7 ± 229.7, median: 235, range: 9.4–1645
SCL-90-R GSI	mean: 0.8 ± 0.6, median: 0.6, range: 0–3.6
GHQ-12 score	mean: 13.6 ± 6.4, median: 12, range: 0–36

^†^Smoke: no, yes (%).

^‡^Psychoactive drugs: no, yes (%).

^§^Opioid agonist: methadone, buprenorphine (%). OMMED: oral morphine milligram equivalent dosage. SCL-90-R GSI: Symptom Check List-90-R global severity index. GHQ-12: 12-Items General Health Questionnaire.

According to IIEF-5 score, 96 patients (12.0%) were sexually inactive, 22 (2.8%) had severe ED, 49 (6.1%) had moderate ED, 86 (10.8%) had mild to moderate ED, 136 (17.1%) had mild ED, and 408 (51.2%) reported no ED. In sexually active patients, average IIEF-5 score was 20.4 ± 5.4 (median: 23, range: 5–25). Only 19 patients (2.4% of the whole sample, 6.4% of sexually active patients with any ED) were under treatment for ED that was prescribed by the Drug Addiction Unit physicians in 4 of them. None of them was treated with androgen replacement therapy.

Patients were grouped into sexually inactive ones (N = 96), those with any ED (i.e., mild to severe, IIEF-5 score < 22, N = 293), and those with no ED (i.e., IIEF-5 score ≥ 22, N = 408).

Age significantly differed according to the group, in that it was larger in sexually inactive patients (42.9 ± 11.3 years) than those with any ED (39.4 ± 9.5) and those with no ED (37.2 ± 9.5; *p* < 0.001), and all post-hoc comparisons were significant between the three groups (Table 3).

Employment status was significantly different between groups (*p* = 0.034), and post-hoc showed a significantly larger number of employed patients (58.1%) in the no ED group than the sexually inactive one (44.8%; Table 3). Smoke was significant between groups (*p* = 0.01), and post-hoc comparison showed a significantly larger number of smokers in the any ED group (15.0%) in comparison to the other groups. The number of cigarettes/day was significantly different across groups (*p* = 0.004), in that it was larger in the sexually inactive group (17.1 ± 8.5) than in the any ED group (13.9 ± 8.6; Table 3).

**Table 3:** Demographic and clinical characteristics of the patients according to the presence of erectile dysfunction (ED).

Variable	Sexually inactive (N = 96)	Any ED (N = 293)	No ED (N = 408)	*P*
Demographic				
Age (y)	42.9 ± 11.3*	39.4 ± 9.5*	37.2 ± 9.5*	<0.001
Education^†^	65 (67.7%), 30 (31.3%), 1 (1.0%)	179 (61.1%), 105 (35.8%), 9 (3.1%)	249 (61.0%), 148 (36.3%), 11 (2.7%)	n.s.
Employment^‡^	53 (55.2%), 43 (44.8%)*	142 (48.5%), 151 (51.5%)	171 (41.9%), 237 (58.1%)*	0.034
Marital status^§^	84 (87.5%), 12 (12.5%)	245 (83.6%), 48 (16.4%)	340 (83.3%), 68 (16.7%)	n.s.
Parenthood^¶^	77 (80.2%), 19 (19.8%)	257 (87.7%), 36 (12.3%)	347 (85.0%), 61 (15.0%)	n.s.
Clinical				
Smoke^¶^	6 (6.3%), 90 (93.7%)	44 (15.0%), 249 (85.0%)**	36 (8.8%), 372 (91.2%)	0.01
Cigarettes/day	17.1 ± 8.5*	13.9 ± 8.6*	15.6 ± 9.6	0.004
Psychoactive drugs^¶^	53 (55.2%), 43 (44.8%)	165 (56.3%), 128 (43.7%)	284 (69.6%), 124 (30.4%)***	<0.001
Psychoactive drug type^ß^				0.001***
Antidepressants	9 (9.4%)	25 (8.5%)	23 (5.6%)	n.s.
Benzodiazepines	17 (17.7%)	68 (23.2%)*	63 (15.4%)*	0.007
Neuroleptics	2 (2.1%)	12 (4.1%)*	6 (1.5%)*	0.034
More than one	15 (15.6%)*	24 (8.2%)	31 (7.6%)*	0.019
Opioid agonist^#^	77 (80.2%), 19 (19.8%)	219 (74.7%), 74 (25.3%)	302 (74.0%), 106 (26.0%)	n.s.
Daily OMMED (mg)	310.2 ± 229.0*	291.3 ± 218.2	270.1 ± 237.5*	0.032
GHQ-12 score	15.3 ± 6.7	14.3 ± 6.5	12.7 ± 6.0**	<0.001

^†^Education: grade school, high school, university.

^‡^Employment: unemployed, employed.

^§^Marital status: single/divorced, engaged/married. ^¶^Parenthood, smoke, psychoactive drugs: no, yes. ^ß^Psychoactive drug type: number of patients (%).

^#^Opioid agonist: methadone, buprenorphine (%).

*Significant difference between groups to post-hoc comparison.

**Significant difference vs. the other two groups to post-hoc comparison.

***No ED group significantly different from the other two groups to post-hoc comparison. OMMED: oral morphine milligram equivalent dosage.

GHQ-12: 12-Items General Health Questionnaire.

The use of psychoactive drugs significantly differed across groups (*p* < 0.001), and post-hoc showed that it was smaller in the no ED group (30.4%) in comparison to the other ones. The type of psychoactive drugs also differed across groups (*p* = 0.001), in that post-hoc showed significant difference between the no ED group and the other ones. Among psychoactive drugs, the use of benzodiazepines (*p* = 0.001), neuroleptics (*p* = 0.034), and more than a single active principle (*p* = 0.019) significantly differed between groups (Table 3). Daily OMMED was significant between groups (*p* = 0.032), and post-hoc showed that it was smaller in the no ED group (270.1 ± 237.5) than in the sexually inactive one (310.2 ± 229.0; Table 3).

GHQ-12 score significantly differed across groups (*p* < 0.001), in that it was smaller (i.e., better QoL) in the no ED group than the other ones.

The other demographical and clinical variables were not significant between groups (Table 3).

**Fig 1:**
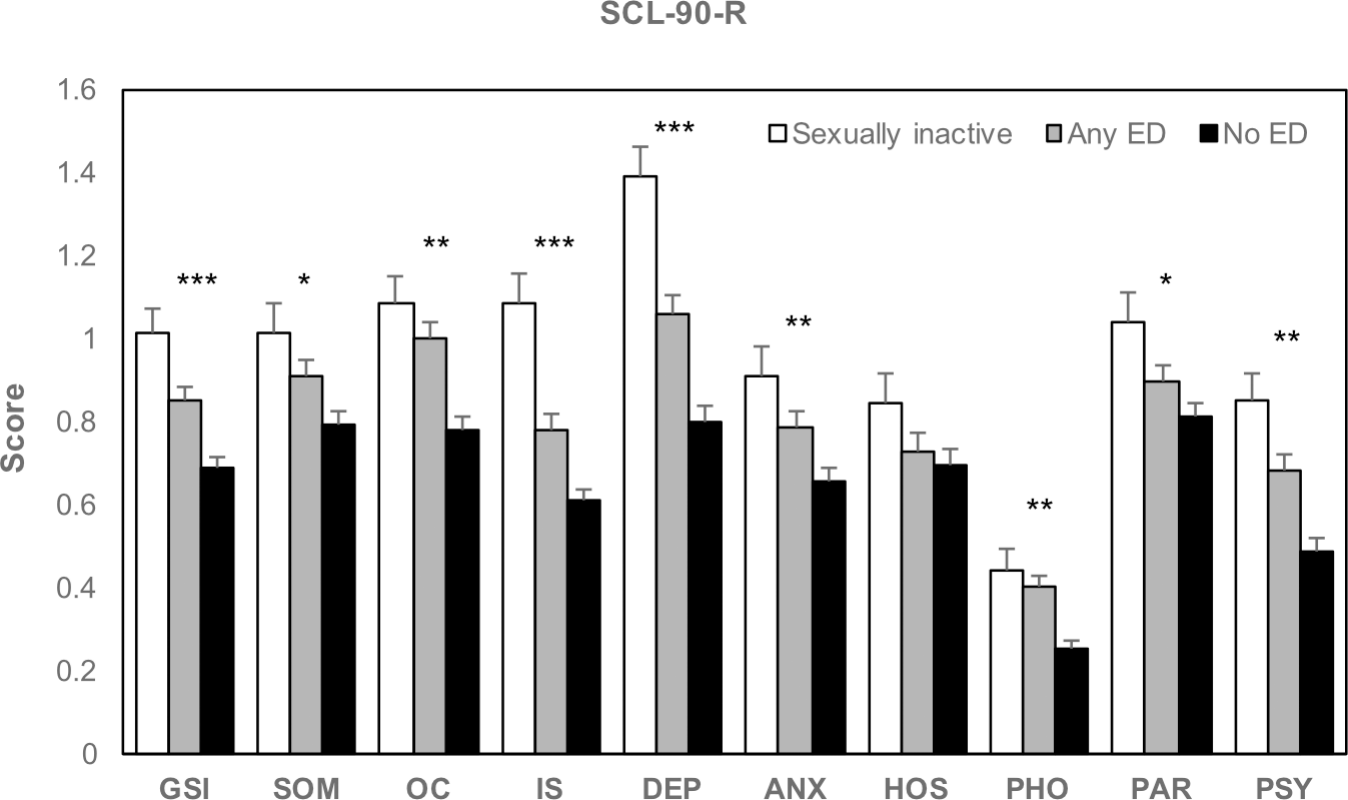
**Psychopathological symptoms according to the Symptom Check List-90-R.** SCL-90-R: Symptom Check List-90-R. GSI: global severity index. SOM: somatization. OC: obsessive-compulsive. IS: interpersonal sensitivity. DEP: depression. ANX: anxiety. HOS: hostility. PHO: phobic anxiety. PAR: paranoid ideation. PSY: psychoticism (PSY). ED: erectile dysfunction. *Significant sexually inactive vs. no ED groups post-hoc comparison (*p* < 0.016). **No ED group significantly different from the two other groups to post-hoc comparison (*p* < 0.016). ***All post-hoc comparisons were significant (*p* < 0.016).

SCL-90-R GSI was significantly different across groups (*p* < 0.001), and all post-hoc comparisons were significant (Fig 1). All SCL-90-R subscales, except HOS (n.s.) were significantly different (SOM: *p* = 0.005, OC: *p* < 0.001, IS: *p* < 0.001, DEP: *p* < 0.001, ANX: *p* < 0.001, PHO: *p* < 0.001, PAR: *p* = 0.013, PSY: *p* < 0.001). Post-hoc showed significant difference in sexually inactive vs. no ED groups comparison for all subscales except HOS, in any ED vs. no ED groups comparison for OC, IS, DEP, ANX, PHO and PSY subscales, and in sexually inactive vs. any ED groups comparison for IS and DEP (Fig 1).

Age (ρ = -0.18, *p* < 0.001) and daily OMMED (ρ = -0.08, *p* = 0.025) were significantly and negatively correlated with the IIEF-5 score, while no significant correlation was found between the number of cigarettes/day and IIEF-5 score. A significant negative correlation was found between IIEF-5 score and GHQ-12 score (ρ = -0.18, *p* < 0.001).

For sexually active patients (N = 701), covariates, which turned out significant in previous between-groups analyses (i.e., age, employment, smoke, cigarettes/day, psychoactive drug, psychoactive drug type, daily OMMED, all SCL-90-R subscales except HOS) were entered into multivariate analysis. Multivariate logistic regression model showed that psychoactive drugs and some SCL-90-R subscales (i.e., DEP, ANX, PHO, PSY) had significant direct effect, and the number of cigarettes/day had significant inverse effect on the risk of developing any ED, while the other covariates were not significant (Table 4). Linear regression model showed that employment, smoke, and some SCL-90-R subscales (i.e., DEP, ANX, PAR, PSY) had significant positive effect, while age, psychoactive drugs, and daily OMMED had significant negative effect on IIEF-5 score (Table 5).

**Table 4:** Logistic regression model analysis for the risk of developing any erectile dysfunction (ED).

Significant covariates	OR	95% CI	*P* value
Cigarettes/day	0.97	0.96; 0.99	0.002
Psychoactive drugs^†^	1.62	1.17; 2.24	0.003
SCL-90-R subscales			
DEP	2.25	1.43; 3.53	<0.001
ANX	2.30	1.59; 3.35	0.005
PHO	1.89	1.13; 3.12	0.015
PSY	1.70	1.02; 2.82	0.041

Here are reported only covariates that turned out to be significant in multivariate logistic regression analysis. OR: odds ratio. CI: confidence interval.

^†^Psychoactive drugs: 0 = no, 1 = yes. SCL-90-R: Symptom Check List-90-R. DEP: depression. ANX: anxiety. PHO: phobic anxiety. PSY: psychoticism.

**Table 5:** Linear regression model analysis for the IIEF-5 score.

Significant covariates	β	95% CI	*P* value
Age (y)	-0.23	-0.20; -0.27	<0.001
Employment^†^	3.84	2.87; 4.81	<0.001
Smoke^‡^	7.87	6.58; 9.17	<0.001
Psychoactive drugs^¶^	-2.03	-4.13; -0.36	0.035
Daily OMMED (mg)	-0.03	-0.01; -0.05	0.003
SCL-90-R subscales			
DEP	-1.79	-3.18; -0.30	0.019
ANX	-2.11	-3.79; -0.44	0.014
PAR	-2.03	-3.12; -0.94	<0.001
PSY	-2.60	-4.15; -1.04	0.001

Here are reported only covariates that turned out to be significant in multivariate linear regression analysis. Please note that higher IIEF-5 scores indicated better erectile dysfunction.

^†^Employment: 0 = unemployed, 1 = employed.

^‡^Smoke: 0 = no, 1 = yes.

^¶^Psychoactive drugs: 0 = no, 1 = yes. IIEF-5: International Index of Erectile Function. CI: confidence interval. OMMED: oral morphine milligram equivalent dosage. SCL-90-R: Symptom Check List-90-R. DEP: depression. ANX: anxiety. PAR: paranoid ideation. PSY: psychoticism.

Multivariate linear regression model was applied to explore the influence of demographic (age, education, marital status, parenthood), clinical variables (type of opioid, daily OMMED, smoke, cigarettes, psychoactive drugs, type of psychoactive drug), SCL-90-R GSI, and IIEF-5 score on GHQ-12 scores in sexually active patients (N = 701). Age, education, daily OMMED, IIEF-5 score, and SCL-90-R GSI significantly worsened, while employment significantly ameliorated QoL according to GHQ-12 score (Table 6).

**Table 6:** Linear regression model analysis for the GHQ-12 score.

Significant covariates	β	95% CI	*P* value
Age (y)	0.13	0.10; 0.16	0.014
Education^†^	0.73	0.12; 1.33	0.02
Employment^‡^	-1.02	-1.76; -0.28	0.007
Daily OMMED (mg)	0.02	0.00; 0.03	0.042
IIEF-5 score	-0.83	-1.34; -0.31	0.002
SCL-90-R GSI	7.47	6.85; 8.08	<0.001

Here are reported only covariates that turned out to be significant in multivariate linear regression analysis. Please note that higher GHQ-12 score indicates worse quality of life.

^†^Education: 0 = grade school, 1 = high school, 2 = university.

^‡^Employment: 0 = unemployed, 1 = employed. GHQ-12: 12-Items General Health Questionnaire. CI: confidence interval. OMMED: oral morphine milligram equivalent dosage. IIEF-5: International Index of Erectile Function. SCL-90-R: Symptom Check List-90-R. GSI: global severity index.

## Discussion

To the best of our knowledge, this is the largest report on ED in men on methadone or buprenorphine maintenance treatment. The main findings, which will be discussed below, were as follows: a) nearly half of patients in our sample were sexually inactive or reported some degree of ED; b) some demographic and clinical variables, as well as most of psychopathological symptoms measured with the SCL-90-R, significantly differed according to the presence or absence of ED; c) multivariate regression model indicated that age, employment, smoke, psychoactive drugs, daily OMMED and some SCL-90-R subscales significantly influenced the risk and severity of ED; d) QoL was worse in patients with ED and significantly correlated with ED severity; e) age, education, employment, daily OMMED, IIEF-5 score, and SCL-90-R GSI significantly influenced QoL in the multivariate analysis.

The 36.8% prevalence of any ED in our sample is in line with previous studies [7,9,11]. Among our patients, 12% were sexually inactive, but we have no information whether this was caused by the severity of ED, or by other factors.

Despite the severity of ED was reported to range from mild to moderate in many patients, its impact was relevant, given the relatively young age of our sample (mean age: 38.6 years). Moreover, the possibility that some patients reported less severe ED symptoms because they felt uncomfortable or embarrassed should be considered [29].

Age was significantly higher in patients with ED and significantly correlated with ED severity (i.e., the higher the age, the more severe the ED) in multivariate analysis. This finding is in keeping with those in opioid users [9,10,13,30], and in the general population [2, 4].

Being employed was found to be associated with reduced severity of ED in our study. This factor was either not consistently associated with ED, or seldom explored in previous studies. Since a direct cause-effect relationship between employment and sexual dysfunction is difficult to hypothesize, we may speculate that psychological and social consequences of being employed might have reduced the severity of ED in our patients.

At variance from previous reports [6,7], marital status did not turn out to be associated with ED in the present study. Differences between the samples or cross-cultural differences [6] might have contributed to this finding.

The effect of smoke appears to be complex in our group of patients. Smoking was more frequent in patients with ED, and was a significant predictor of ED severity in multivariate analysis (i.e., more severe ED in smokers). The number of daily cigarettes turned out to be significantly higher in sexually inactive patients than those with ED, and a significant predictor of any ED in logistic multivariate analysis (i.e., the smaller the number of cigarettes/day, the higher the risk of ED). While smoke stands amongst the main factors associated with ED in general population [2,4], the role of this factor was unclear in previous studies on men using opioids [10,13,30]. Smoke, which is common in people using opioids [31], may contribute to ED through increase in oxidative stress and inflammatory markers, that bring about vascular changes [32]. Having been the assessment of this factor based on self-report in our study, a recall (i.e., patients did not precisely remember the number of daily cigarettes) or response bias (i.e., patients reported a smaller number of daily cigarettes) might have contributed to the apparently contrasting finding that the number of cigarettes/day was inversely associated with the risk of ED. Moreover, smokers often vary their smoking habits over the years, and the use of smoking pack-years instead of daily cigarettes would have represented a better measure of lifetime tobacco exposure in our patients.

The use of other psychoactive drug was less common in patients with no ED than those with any ED or who were sexually inactive, and this factor was significant predictor for the risk of any ED and its severity. Benzodiazepines and neuroleptics were found to be more frequent in patients with ED than those with no ED, and the use of more than one class of psychoactive drugs was more frequent in sexually inactive patients than those with no ED. This finding, which is in accordance with previous studies [13,33], is not surprising, since psychoactive drugs may have negative effects on sexual function through various mechanisms. Moreover, the coexisting psychopathology might contribute to ED through psychological mechanisms. Of interest, antidepressants, which were reported to be associated with sexual dysfunction in previous reports [30,34], did not differ across groups in our sample.

We did not find significant difference between methadone and buprenorphine treatment. Previous studies reported contrasting results on this point [7–9,19,20], with a meta-analysis indicating that methadone has 4.01 odds ratio (95% CI: 1.52–10.55) for sexual dysfunction in comparison to buprenorphine [13]. Reasons for previous contrasting data might include the small number of patients, and/or the absence of multivariate analysis to correct for potential confounders.

Daily OMMED significantly differed between groups, being larger in sexually inactive patients, intermediate in patients who reported any ED, and smaller in those with no ED. Moreover, daily OMMED was significantly correlated with the IIEF-5 score (i.e., the larger the dosage, the more severe the ED), and the correlation survived multivariate analysis. Data from previous studies are conflicting on this point, as some of them found significant correlation between opioid dosage and sexual dysfunction [35,36], while other ones reported negative findings [6,7]. It is tempting to speculate that higher opioid dosage could have resulted in more marked effects on hypothalamic–pituitary axis that in turn might have been responsible for ED in our patients.

Nearly all SCL-90-R subscales were significantly different across groups, being more severe in sexually inactive patients than any ED and no ED groups. For some subscales, a significant difference was found when comparing patients with ED to those without ED. Five SCL-90-R subscales (i.e., DEP, ANX, PHO, PAR, PSY) were significant predictor of either the risk of ED or its severity in multivariate analysis. These data, which are in accordance with previous studies in the general population with no medical problems [5], and in patients receiving opioid maintenance treatment [7,37], underscore the importance of comorbid psychopathology in patients with ED. The association might be bidirectional, because psychological factors may result in ED that, in turn, may worsen psychopathology [7]. Future longitudinal studies would be important to better explore the directionality of the cause-effect relationship between psychological comorbidity and ED.

Despite the recommendation that androgen replacement therapy should be considered in males diagnosed with OPIAD [17], none of our patients were on this treatment. A small minority of them (i.e., 6.4% of sexually active patients with any ED) received treatment for ED. These figures appear to differ from those in patients under long-term opioids for back pain, 19% of whom were reported to receive prescription for ED or androgen replacement therapy, especially in case of high dosage and longer duration of opioid treatment [38]. Reasons for this negative finding in our sample might include concern on the possible cardiovascular and hepatic side effects, the risk of prostate cancer, or the lack of awareness for this therapy by the treating physicians and/or patients.

QoL was significantly worse in patients who were sexually inactive or who reported ED in comparison to those with no ED. A significant correlation was found between IIEF-5 and GHQ-12 scores, and multivariate analysis showed that ED score was a predictor of QoL impairment (i.e., the worse the ED, the worse the QoL). The other factors that influenced QoL severity were age, education, employment, daily OMMED, and SCL-90-R GSI, most of which were found to significantly influence ED. It is not surprising that ED may impact on QoL through impairment of physical and psychological functioning, but surprisingly this outcome was seldom explored in men under ORT [6,8,21,22].

The first and most important limitation of the present study is the lack of data on testosterone or hypogonadism, but previous studies reported no correlation between testosterone levels and ED severity, especially in younger men, probably because serum levels do not reflect biologically available testosterone, a more accurate marker of hypogonadism [9,30]. The second limitation is that, because of the large sample in the present study, the assessment of ED was subjective and patients did not undergo objective measures, which could have helped better separate physical and psychological determinants of ED. The third limitation is possible response bias, since some patients might have concealed or reported a smaller severity of their symptoms, because they felt uncomfortable or embarrassed when answering questions on ED [8]. This hypothesis is strengthened by the relatively large number of patients (nearly 20%), who preferred not to answer the IIEF-5 questionnaire and were not included in the study. Other limitations include the cross-sectional nature of the study, and the lack of information on coexisting viral infections, such as viral hepatitis or HIV. Hepatitis C virus infection, a common comorbid condition in opioid users, can negatively affect sex hormone levels, but its role in ED is still contradictory [39,40].

Our data showed that nearly half of our patients under ORT reported ED or were sexually inactive, ED severity was significantly correlated to QoL, and cigarette smoking and use of psychotropic drugs was predictive of ED. Sexuality is still a neglected issue in people with substance use disorders, but sexual and ED may have important effects on emotional and social well-being, including self-esteem and sense of self-efficacy, and may affect outcome by improving adherence to treatment [8]. Exploring ED complaints in men using opioids under ORT is a critical component of care, because self-report on this problem still occurs in a minority of patients [41], and IIEF-5 represents a simple and quick self-assessment tool. Counselling patients about the importance of smoking cessation is paramount especially in this population. Future studies should explore whether reducing ED symptoms in men under ORT might improve QoL, and reduce relapse into opioid use.

## Supporting information

S1 Dataset Dataset of the study. The dataset of the study with demographic and clinical data of the patients.
(XLS)
